# Verbal and visuospatial short-term and working memory data across a 43-year period (1973–2016) worldwide: Flynn and anti-Flynn effects

**DOI:** 10.1016/j.dib.2020.105231

**Published:** 2020-02-03

**Authors:** Peera Wongupparaj, Rangsirat Wongupparaj, Veena Kumari, Robin G. Morris

**Affiliations:** a Cognitive Science and Innovation Research Unit (CSIRU), College of Research Methodology and Cognitive Science, Burapha University, Thailand; b Department of Psychology, King's College London, Institute of Psychiatry, Psychology, and Neuroscience, UK; c Department of Curriculum and Instruction, Faculty of Education, Chulalongkorn University, Thailand; d Centre for Cognitive Neuroscience, College of Health and Life Sciences, Brunel University London, UK

**Keywords:** Flynn and anti-Flynn effects, A cross-temporal meta-analysis, Forward and backward digit span tests, Forward and backward Corsi-block span tests, Verbal and visuospatial short-term and working memory

## Abstract

Secular gain and drop in cognitive test performances over time have been observed and called respectively the Flynn and anti-Flynn effects. The current datasets include raw data from an investigation of the Flynn and/or anti-Flynn effects on verbal and visuospatial short-term and working memory reported in ‘The Flynn effect for verbal and visuospatial short-term and working memory: A cross-temporal meta-analysis’ (Wongupparaj, Wongupparaj, Kumari, Morris, 2017) [1]. Specifically, the datasets totally contain 1754 individual samples (n = 139,677) across a 43-year period from forward/backward digit span (F/BDS) and forward/backward Corsi-block span (CBS) tests. Mean memory test scores, standard deviation scores, types of memory tests, years of publication, mean ages, male percentages, types of publication, types of countries, platforms of memory tests, and sample sizes were collected and included in the datasets. DS and CBS data are unique in that they can provide a rich source of trends concerning changing short-term and working memory test scores across memory types, test platforms, age groups, gender, and countries. Further, these data can be of use for investigation of psychometric properties for the memory tests.

**Table:** Specifications Table

Subject	Psychology
Specific subject area	Applied Psychology; Experimental and Cognitive Psychology; Psychology (General)
Type of data	Sav files (SPSS files)
How data were acquired	The data were reviewed and collected from the research articles and theses/dissertations published in English, obtained using the PRISMA guideline for conducting and reporting literature searches [2].
Data format	Raw
Parameters for data collection	The mean and/or standard deviation scores for memory tests (i.e. F/B DS and F/B CBS) from each study were collected and included in the final datasets. In addition, types of memory tests, the authors, years of publication, mean ages, male percentage, types of literature (journal versus thesis/dissertation), types of countries (developing and developed), platforms of memory tests (computer-delivered and paper-based or wooden cube-based), and sample sizes were also incorporated in the final datasets.
Description of data collection	All target studies and parameters were identified through 15 scientific databases from 1910 to 2016, namely BioMed Central, BMJ, Cambridge Journals, Frontiers in Psychology, HighWire Journal, JSTOR, Karger Online Journals, Oxford Journals, ProQuest dissertations and theses, PsycARTICLES & PsychINFO, Sage Journals, ScienceDirect, SpringerLink, Taylor & Francis Online, and Wiley Online Library. All samples were reviewed and assessed in line with adopted inclusion and exclusion criteria (see ‘Experimental designs, Materials, and Methods’).
Data source location	The F/B DS and F/B CBS datasets contain target parameters derived from published and unpublished studies and developing and developed countries worldwide.
Data accessibility	Repository name: Mendeley data Data identification number: https://doi.org/10.17632/t64gnmbfk3.1 Datasets can be accessed at Mendeley data: Wongupparaj, Peera (2019), “Datasets for forward/backward digit span and Corsi-block tests covering 43 years (1973–2016)”, Mendeley Data, V1, https://doi.org/10.17632/t64gnmbfk3.1 The direct URL to data: https://doi.org/10.17632/t64gnmbfk3.1
Related research article	**Author's name:** Wongupparaj, P., Wongupparaj, R., Kumari, V., & Morris, R. G. **Title:** The Flynn effect for verbal and visuospatial short-term and working memory: A cross-temporal meta-analysis **Journal:** Intelligence **DOI:**https://doi.org/10.1016/j.intell.2017.07.006

**Table:** 

**Value of the Data** • The dataset includes an extensive overview of the key variables for two versions (i.e. forward and backward) of F/B DS and F/B CBS tests across years of publication, mean ages, types of countries, male percentage, sample sizes, types of literature, and platforms of the memory tests. • The data may be used for constructing the test norms and testing the psychometric properties (i.e. validity and reliability) of F/B DS and F/B CBS tests. • The obtained data can be used for estimating the Flynn and anti-Flynn effect on test interpretation across the additionally provided variables. • The datasets will be of use to investigate changes on verbal and visuospatial short-term and working memory scores overtime and also provide a beginning point for conducting further meta-analysis or computing any updated meta-analytic estimates from 1973 to the present [1].

## 1 Data

The datasets consist of four SPSS.sav files that contains key parameters for FDS, BDS, FCBS, and BCBS tests. All four datasets generally share a similar structure and number of contained variables, that is, authors of each sample, years of publication, mean ages of each sample, male percentage in each sample, types of literature, types of country, tests platform, age groups, sample sizes, mean scores of memory tests, standard deviation scores of memory tests, standard error scores of memory tests, and variance scores of memory tests.

The FDS dataset specifically includes 13 variables from 742 independent samples with a total of 48,955 people. The 13 variables show authors of each sample, years of publication (1973–2016), mean ages of each sample (Min-Max = 3.65 to 92.40), male percentage in each sample (M = 44.98%; Min-Max = 0%–100%), types of literature (92.30% journals and 7.70% dissertations), types of country (9.30% developing and 90.70% developed), tests platform (8.90% computer-delivered and 91.10% paper-based or wooden cube-based), age groups (10.20% 3–6 years old, 18.40% 7–12 years old, 22.90% 13–25 years old, 10.60% 26–39 years old, 7.50% 40–60 years old, and 30.50% more than 60 years old), sample sizes (1–4100 people), mean scores of memory tests (0.81–9.10), standard deviation scores of memory tests (0–3.08), standard error scores of memory tests (0–0.79), and variance scores of memory tests (0–9.49).

The BDS dataset also contains 13 variables from 594 independent samples with a total of 70,424 people. The 13 variables consist of authors of each sample, years of publication (1975–2016), mean ages of each sample (Min-Max = 4 to 92.50), male percentage in each sample (M = 45.38%; Min-Max = 0%–100%), types of literature (92.40% journals and 7.60% dissertations), types of country (10.90% developing and 89.10% developed), tests platform (7.10% computer-delivered and 92.90% paper-based or wooden cube-based), age groups (8.30% 3–6 years old, 20.40% 7–12 years old, 19.60% 13–25 years old, 7.80% 26–39 years old, 9.80% 40–60 years old, and 34.10% more than 60 years old), sample sizes (5–4251 people), mean scores of memory tests (1.08–7.20), standard deviation scores of memory tests (0.10–3.50), standard error scores of memory tests (0.01–1.06), and variance scores of memory tests (0.01–12.25).

The FCBS dataset consists of 13 variables from 307 independent samples with a total of 16,514 people. The 13 variables include authors of each sample, years of publication (1982–2016), mean ages of each sample (Min-Max = 3.04 to 86.65), male percentage in each sample (M = 46.96%; Min-Max = 0%–100%), types of literature (98.70% journals and 1.30% dissertations), types of country (7.80% developing and 92.20% developed), tests platform (25.40% computer-delivered and 74.60% paper-based or wooden cube-based), age groups (11.40% 3–6 years old, 23.10% 7–12 years old, 26.40% 13–25 years old, 13.70% 26–39 years old, 6.50% 40–60 years old, and 18.90% more than 60 years old), sample sizes (9–632 people), mean scores of memory tests (2.20–7.80), standard deviation scores of memory tests (0.10–3.00), standard error scores of memory tests (0.02–0.66), and variance scores of memory tests (0.01–9.00).

Finally, the BCBS datasets offers 13 variables from 111 independent samples with a total of 3784 people. The 13 variables include authors of each sample, years of publication (1989–2016), mean ages of each sample (Min-Max = 4.00 to 86.65), male percentage in each sample (M = 46.22%; Min-Max = 0%–100%), types of literature (98.70% journals and 1.30% dissertations), types of country (7.80% developing and 92.20% developed), tests platform (25.40% computer-delivered and 74.60% paper-based or wooden cube-based), age groups (11.40% 3–6 years old, 23.10% 7–12 years old, 26.40% 13–25 years old, 13.70% 26–39 years old, 6.50% 40–60 years old, and 18.90% more than 60 years old), sample sizes (9–632 people), mean scores of memory tests (2.10–7.09), standard deviation scores of memory tests (0.17–2.53), standard error scores of memory tests (0.03–0.52), and variance scores of memory tests (0.03–6.40).

A complete list of references for included sample and studies across the four memory tests is openly available at https://osf.io/3wa94/

## 2 Experimental design, materials, and methods

### 2.1 Data and literature search strategy

The search terms used were “digit span”, “Wechsler's digit span”, “forward digit span”, “backward digit span”, “Corsi block*” (* represents multiple spellings and endings), “forward Corsi block”, and “backward Corsi block”. These search terms were employed individually and in combination with the Boolean OR function in order to increase search sensitivity. The target literatures were systematically searched through 15 scientific databases that contained journals and/or theses and dissertations (unpublished materials or grey literature) from 1910 to 2016. The following scientific databases were BioMed Central, BMJ, Cambridge Journals, Frontiers in Psychology, HighWire Journal, JSTOR, Karger Online Journals, Oxford Journals, ProQuest dissertations and theses, PsycARTICLES & PsychINFO, Sage Journals, ScienceDirect, SpringerLink, Taylor & Francis Online, and Wiley Online Library. All search outputs from databases were exported to a reference management software, EndNote, and duplicates were then removed.

### 2.2 Inclusion and exclusion criteria

The final datasets for all memory tests were only from studies which reported the mean and/or standard deviation of raw scores from FDS, BDS, FCBS, and BCBS. Thus, samples were excluded if they reported the target parameters for age-scaled and standardized scores. Further, only mean and/or standard deviation scores and relevant variables for pre-test or baseline were collected if these studies adopted a test-retest method or a time series analysis. In addition, only mean and/or standard deviation scores and relevant variables were treated as a single data point for multiple studies with the same sample or shared datasets. Also, studies with clinical research participants were excluded with the exception if they reported data for health controls or control groups. The following samples were excluded because no target parameters were reported; book chapters, review articles, systematic reviews, meta-analyses, research protocols, and case reports.

Finally, outlier and influential case diagnostics were performed on all target parameters across all studies using Cook's distances, DFBETAS, and DFFITS. Accordingly, final datasets include 742, 594, 307, and 111 independent samples for FDS, BDS, FCBS, and BCBS respectively, resulting in 139,667 participants overall.

### 2.3 Data extraction

Two research assistants independently reviewed all relevant parameters across samples. The process of identification, screening, and eligibility check for including target parameters in the final datasets was conducted according to the PRISMA guideline [2].
